# Describing Unmet Supportive Care Needs among Young Adults with Cancer (25–39 Years) and the Relationship with Health-Related Quality of Life, Psychological Distress, and Illness Cognitions

**DOI:** 10.3390/jcm10194449

**Published:** 2021-09-28

**Authors:** Emma Lidington, Anne-Sophie Darlington, Amy Din, Susannah Stanway, Susana Banerjee, Zoltan Szucs, Michael Gonzalez, Anand Sharma, Bhawna Sirohi, Winette T. A. van der Graaf, Olga Husson

**Affiliations:** 1 The Royal Marsden NHS Foundation Trust, 203 Fulham Road, London SW3 6JJ, UK; emma.lidington@rmh.nhs.uk (E.L.); susannahstanway@gmail.com (S.S.); Susana.banerjee@rmh.nhs.uk (S.B.); w.vd.graaf@nki.nl (W.T.A.v.d.G.); 2 Health Sciences, University of Southampton, University Road, Highfield, Southampton SO17 1BJ, UK; a.darlington@soton.ac.uk (A.-S.D.); aed3g11@soton.ac.uk (A.D.); 3 Division of Clinical Studies, Institute of Cancer Research, 15 Cotswold Road, Sutton SM2 5NG, UK; 4 East Suffolk and North Essex NHS Foundation Trust, Ipswich Hospital, Health Road, Ipswich IP4 5PD, UK; z.szucs@nhs.net; 5 Imperial College Healthcare NHS Trust, Charing Cross Hospital, Fulham Palace Road, Hammersmith, London W6 8RF, UK; Michael.gonzalez@icr.ac.uk; 6 Mount Vernon Cancer Centre, Rickmansworth Road, Northwood HA6 2RN, UK; anand.sharma3@nhs.net; 7 Barts Health NHS Trust, St Bartholomew’s Hospital, W Smithfield, London EC1A 7BE, UK; bhawna.sirohi13@gmail.com; 8 Apollo Proton Cancer Centre, 4/661, Dr Vikram Sarabai Instronic Estate 7th St., Dr. Vasi Estate, Phase II, Tharamani, Chennai 600096, India; 9 Department of Medical Oncology, Netherlands Cancer Institute–Antoni van Leeuwenhoek, Plesmanlaan 121, 1066 CX Amsterdam, The Netherlands

**Keywords:** supportive care, young adults, AYA, cancer, quality of life

## Abstract

Few studies describe supportive care needs among young adults (YAs) with cancer ages 25 to 39 using validated questionnaires. Previous findings identified the need for psychological and information support and suggest that gender, age, psychological distress, and coping may be associated with greater need for this support. To substantiate these findings, this study aimed to (1) describe the supportive care needs of YAs in each domain of the Supportive Care Needs Survey and (2) explore the relationship between unmet supportive care needs and clinical and demographic factors, health-related quality of life, psychological distress, illness cognitions, and service needs using latent class analysis. Clinical teams from six hospitals in England invited eligible patients to a cross-sectional survey by post. A total of 317 participants completed the survey online or on paper. YAs expressed the most need in the psychological and sexuality domains. Using latent class analysis, we identified three classes of YAs based on level of supportive care need: no need (53.3%), low need (28.3%), and moderate need (18.4%). In each class, median domain scores in each domain were similar. Low and moderate need classes were associated with worse health-related quality of life and greater helplessness. Unmet service needs were associated with the moderate-need class only. Patients with unmet supportive care needs should be offered holistic care across supportive care domains.

## 1. Introduction

A growing body of research has highlighted specific psychosocial issues experienced by young adults (YAs) ages 25 to 39 with cancer, such as difficulty balancing work or childcare with treatment, financial distress, and social isolation from friends and family [1,2]. However, evidence on whether YAs need access to support services for the issues experienced is lacking. In cancer, supportive care refers to ‘the provision of the necessary services for those living with or affected by cancer to meet their informational, emotional, spiritual, social, or physical needs during their diagnostic, treatment, or follow-up phases encompassing issues of health promotion and prevention, survivorship, palliation, and bereavement’ [3]. Simply measuring the prevalence or severity of problems assumes that patients who experience issues have a need for supportive care. Needs assessments directly measure if a patient perceives a need for help and the magnitude of the desire for help [4].

Three systematic reviews including qualitative and quantitative studies have looked at supportive care needs among adolescents and YAs and identified a need for age-appropriate information, facilities, and communication, access to emotional support, contact with peers, and fertility information and services [5,6,7]. However, many studies used qualitative data, and few quantitative studies used validated measures of need. Additionally, most studies focused on younger patients ages 15 to 24, who have better access to age-tailored psychosocial support in the countries where the studies were conducted.

One more recent study included adolescents and YAs ages 18 to 39 and used the Supportive Care Needs Survey (SCNS). The SCNS is a validated measure comprised of common issues among cancer patients in five domains of need: psychological, health system and information, patient care and support, physical and daily living, and sexuality needs [8]. Met needs are the issues that patients report as not applicable or ‘satisfied’, while unmet needs are the issues where patients report they have some degree of need. This study found the highest unmet supportive care needs were in the psychological and information domains. Higher unmet needs in some domains were associated with female gender, older age, increased distress, and poorer coping with the disease. These findings generally support recent research into adolescent and YA care advocating for more age-appropriate information and psychosocial support [9].

Unmet supportive care needs in previous studies have been associated with lower health-related quality of life (HRQoL) and higher psychological distress [10,11,12,13]. Contrary to expectation, one study exploring the relationship between function, symptoms, and supportive care needs found patients with low function and high symptoms did not always have high unmet supportive care needs [14]. This may reflect variable access to psychosocial support services or differences in cognitive processing. Variable access to support services can lead to ‘service need’ where a patient is unable to use a certain desired service (i.e., psychology or physiotherapy). This differs from supportive care need, which relates to support for specific issues or problems common among patients with cancer (i.e., anxiety or pain). Illness cognitions, the beliefs or perceptions patients have about their disease and its treatment, may be related to a patient’s HRQoL [15]. The relationship between illness cognition, service need, and supportive care need has not yet been explored among YAs with cancer.

To substantiate the unmet supportive care needs of YAs ages 25 to 39 and examine the relationship with clinical and demographic factors and other psychosocial concepts, we conducted a multi-centre cross-sectional survey. Our main objectives were to (1) describe the unmet supportive care needs among YAs in each SCNS domain and (2) explore the relationship between supportive care need and clinical and demographic factors, HRQoL, psychological distress, illness cognitions, and service need using latent class analysis.

## 2. Materials and Methods

### 2.1. Study Design

We conducted a multi-centre, cross-sectional survey.

### 2.2. Study Population and Procedures

Clinical teams from six hospitals across southeast England identified potential participants in clinic lists and local databases. Eligible patients were diagnosed with any cancer type between age 25 and 39 between May 2013 and May 2018. Patients were excluded if previously diagnosed with cancer before age 25 or before May 2013, unable to read or write in English or mentally or physically unfit (e.g., severe cognitive disability or nearing end-of-life) as determined by the clinical team. Eligible patients who relapsed or received a second primary diagnosis were not excluded. The clinical team invited patients by letter to take part in the survey between May 2018 and March 2019. Participants that did not respond within one month were posted a reminder letter. Participants could choose to complete the survey online using PROFILES, a web-based system for collecting patient-reported outcomes in cancer research, or return a paper version by post [16].

All participants completed an informed consent form either online or on paper returned with the survey. The study was reviewed and approved by The Royal Marsden NHS Foundation Trust and Institute of Cancer Research Joint Committee for Clinical Research (CCR4648), a London Research Ethics Committee, and the UK Health Research Authority (17/LO/0219).

### 2.3. Measures

All items and measures in the survey were self-reported. Demographic and clinical items included current age, age at diagnosis, gender, ethnicity, education, cancer diagnosis, treatments, current treatment status, and current treatment intent. Here, anti-hormonal treatments were considered active therapy.

#### 2.3.1. Supportive Care Needs

We used the SCNS long form, which is a 59-item instrument that measures supportive care needs among people with cancer [4]. Each item asks patients about a common issue or problem experienced by patients with cancer that can be potentially ameliorated by supportive care. It is a well-validated measure used extensively in cancer populations. The measure has five domains (psychological, health system and information, physical and daily living, patient care and support, and sexuality needs) and 4 single items that do not belong to a domain (talking to other people, changes in others’ attitudes or behaviour towards you, financial concerns, transport). Items are scored from 1 to 5 (1 not applicable, 2 satisfied, 3 low need, 4 moderate need, and 5 high need). Domain scores are the average score of items in each domain and can range from 1–5. Domain scores were calculated if at least half the items were complete. Participants with missing domain scores were excluded. Then, domain scores for each participant were dichotomised with scores >2 indicating ‘unmet need’ as the scoring manual recommends. At an item level, we dichotomised responses with scores >2 indicating ‘unmet need’ and present the ten most common issues of all 59 items. Cronbach’s alpha was calculated to determine internal reliability in the sample.

#### 2.3.2. Quality of Life

The European Organisation for Research and Treatment of Cancer Quality of Life Questionnaire Core 30 (EORTC-QLQ-C30) is a 30-item instrument developed to assess HRQoL in patients with cancer [17]. The measure has been widely used in clinical trials and has robust psychometric properties. The EORTC-QLQ-C30 includes five functional scales (physical, emotional, cognitive, role, and social functioning), eight symptom scales (fatigue, pain, nausea and vomiting, dyspnoea, insomnia, appetite loss, constipation, and diarrhoea) and a global quality of life score. All domains are scored if at least half the items are complete and are transformed to a standardised score of 0–100 with higher scores indicating better function or quality of life or higher symptom burden. A summary score, which has been shown to be a strong prognostic factor for survival, was calculated using the mean of all scale scores except global quality of life and financial impact following the recommendation by Giesinger et al. [18].

#### 2.3.3. Psychological Distress

The Hospital Anxiety and Depression Scale is a 14-item instrument used extensively in cancer research with robust evidence of validity [19,20]. The measure is comprised of two scales (anxiety and depression) made of seven items each. Items are scored from 0 to 3. Scale scores, the summed item total, can range from 0 to 21. Higher scores indicate worse anxiety or depression. Scores greater than eight indicate borderline abnormal anxiety or depression [19,21].

#### 2.3.4. Illness Cognition

Illness cognitions were measured using the Illness Cognition Questionnaire, an 18-item instrument comprised of three, six-item scales: helplessness, acceptance, and perceived benefits [22]. The helplessness scale measures negative perception of the disease as uncontrollable, unpredictable or unchangeable. Acceptance measures the level that a patient acknowledges the illness and perceives the ability to live with the effects of the condition. Perceived benefits measures the amount a patient finds positive meaning in the disease. Items are scored on a 1–4 scale, and scores are the summed totals ranging between six and 24 with higher scores representing greater helplessness, acceptance, or perceived benefits.

#### 2.3.5. Service Needs

The amount to which YAs were able to use desired support services was measured using a non-validated questionnaire adapted from the Adolescent and Young Adult Health Outcomes and Patient Experience Study [23]. Patients were presented with 16 relevant services including physiotherapy, pain management, psychology, and complementary services (Supplementary Materials Table S1). For each item, patients were asked to indicate if they had needed the service and, if yes, if they had used the service. The number of services that were needed but not used were summed to give the total number of unmet service needs for each participant. The total number of service needs could range from 0 to 16. This is another measure of need that asks patients to explicitly report the need and use of specific services rather than the need for support with issues or problems reported in the SCNS. Using both measures allowed us to explore whether service need is related to supportive care need.

### 2.4. Statistical Analysis

Descriptive statistics were calculated for demographic and clinical items and questionnaires. We compared the characteristics of included and excluded participants to identify potential bias using independent samples *t*-tests in the case of continuous variables and chi-squared tests with adjusted residuals in the case of categorical variables.

All continuous variables are presented using mean and standard deviation or median and interquartile range where skewed. Frequency and percentage are reported for categorical variables. *p*-values were considered significant at the point 0.05 level.

#### 2.4.1. Latent Class Analysis

To explore the pattern of responses across SCNS domains and analyse supportive care need as a single outcome to avoid type I errors, we used latent class analysis [24]. Latent class analysis assumes that one or more unobserved categorical variables are responsible for response patterns, which it uses to probabilistically assign individuals to classes and provide information about how individuals are likely to respond to each of the domains given class assignment. Individuals with similar response patterns will tend to be assigned to the same latent class. Then, researchers assign each class a qualitative description based on the literature, experience, and theory. In a previous study in adults, the authors found that the level of supportive care need categorised cancer patients into three classes: low need, moderate need, and high need [25].

Latent class models with increasing numbers of classes were fit from a 1-class to a 5-class model. Model selection was based on minimising the Bayesian Information Criteria (BIC) and Akaike’s Information Criteria (AIC), increasing the entropy and ease of interpretation (i.e., classes make sense from a rational perspective). We determined that the 3-class model was optimal and defined the latent variable as the level of supportive care need. Classes were labelled no need, low need, and moderate need as responses tended to cluster by similar degree of need across supportive care domains, similar to the previous study in adults. Detailed information regarding model fit and selection can be found in Supplementary Materials S2.

#### 2.4.2. Covariates

The relationships between level of supportive care need and clinical and demographic characteristics, psychosocial factors, and access to services was explored in univariable latent class regression models. Diagnosis was dichotomised (breast vs. non-breast) due to small numbers in non-breast cancer diagnoses groups. Variables significantly associated with the level of supportive care need were added to a multivariable latent class regression model using forward selection. Variables were included in the final model if they reduced the AIC and BIC. Collinearity of covariates was tested in a correlation matrix.

Analysis was conducted in R version 4.0.2.3. Data was collected using PROFILES version 1.0, Tilburg, Netherlands.

## 3. Results

Three hundred and forty-seven YAs took part in the survey of 1683 (20.6%) potential participants. Of the respondents, 317 participants completed at least half of each domain in the SCNS and were included in the analysis. Participants were on average 33.3 years old (SD + 4.2) at diagnosis and 2.9 years from diagnosis (SD + 1.6) (Table 1). Most participants were female (N = 219; 69.1%), white (N = 272; 85.8%), and receiving follow-up care and monitoring but no longer receiving anti-cancer treatment (N = 242; 76.3%). Participants excluded from analysis were no different in age at diagnosis (*t* = 0.58; *p* = 0.560), current age (*t* = 0.56; *p* = 0.578), time from diagnosis (*t* = 0.02; *p* = 0.986), gender (X^2^ = 0.07; *p* = 0.785), or cancer type (X^2^ = 6.49; *p* = 0.592) from those included. However, they were more likely to be from ‘other’ ethnic groups (X^2^ = 15.07; *p* = 0.005; adjusted residual =3.25) or have missing treatment status information (X^2^ = 32.98; *p* < 0.001; adjusted residual =5.66). The majority of respondents (53.3%) were in the no-need class, while 28.3% were in the low-need class, and 18.4% were in the moderate-need class.

### 3.1. Supportive Care Needs

Respondents had the highest need in the psychological domain, where 42.0% of all respondents had unmet need (domain score >2), followed by the sexuality domain, where 36.3% reported unmet need (Table 2). When stratified by latent class, at least 60% of participants in the moderate-need class had unmet need in each domain. This contrasts the no-need class, where less than 12% of patients had unmet need in each domain. Cronbach’s alpha for all domains was at least 0.88, indicating good internal reliability.

At the item level, uncertainty about the future and fear of cancer recurrence (FCR) were the most common unmet needs for all patients regardless of class (Table 3). Even in the no-need class where unmet need was uncommon, one-third of patients reported unmet need for uncertainty about the future, and one-fifth of patients reported unmet need for FCR.

Due to the high domain scores in the sexuality domain, we further explored these single items. Support with changes in sexual feelings was unmet in 76.5% (*n* = 39) of moderate-need patients, 52.0% (*n* = 51) of low-need patients, and 11.0% (*n* = 19) of no-need patients. Support with changes in sexual relationships was unmet for 80.4% (*n* = 41) of moderate-need patients, 49.0% (*n* = 48) of low-need patients, and 11.0% (*n* = 19) of no-need patients.

### 3.2. Covariates

Median and interquartile range of the EORTC-QLQ-C30 summary, anxiety, depression, acceptance, helplessness, and perceived benefits scores and number of unmet service needs are presented in Table 4. For each outcome, the median score was worst in the moderate-need class and best in the no-need class.

Breast vs. non-breast diagnosis, white vs. non-white ethnicity, time from diagnosis, chemotherapy, treatment status, treatment intent, diarrhoea, all other EORTC-QLQ-C30 scale scores, the EORTC-QLQ-C30 summary score, anxiety, depression, helplessness, acceptance, and number of unmet service needs were significantly associated with level of supportive care need in univariate analysis (Table 5). As all EORTC-QLQ-C30 scale scores had a strong association with level of supportive care need, the summary score was added to the multivariable model instead of individual scores.

After forward selection, the final multiple regression model included the EORTC-QLQ-C30 summary score, number of unmet service needs, and acceptance. Compared to patients in the no-need class, patients in the low-need class had significantly lower odds of a higher EORTC-QLQ-C30 summary score and significantly higher odds of a higher helplessness score (Table 6). Compared to patients in the no-need class, patients in the moderate-need class had significantly lower odds of a higher EORTC-QLQ-C30 summary score and significantly higher odds of more unmet service needs. The odds of having higher acceptance were lower in the low- and moderate-need classes compared to the no-need class, but these were not significant.

## 4. Discussion

Our results suggest that about half of YAs with cancer have unmet supportive care needs. Among these patients, the degree of need for help is generally low to moderate. Our results substantiate the common unmet need for psychological support among YAs. Evidence suggests a number of interventions are effective at improving psychological well-being among adolescents and YAs with cancer including peer support, technology-based interventions, and skill-based interventions, which could be implemented to address this gap [26].

The most common psychological issues were uncertainty about the future and FCR. In this study, about half the participants experienced FCR, aligning with previous research that found between 29% and 85% of adolescents and YAs experience FCR to some extent [27]. A recent meta-analysis showed that psychological interventions can have small but significant and sustained effects on FCR, particularly contemporary cognitive behavioural therapies [28]. While interventions for uncertainty, which often include informational support, have shown positive effects, a systematic review found these studies to be at unknown or high risk of bias [29]. Further rigorous research should be conducted to evaluate potential psychological support for uncertainty.

Our results also highlight the common unmet need for support in the sexuality domain. Specifically, respondents reported unmet need for support with changes in sexual feelings and relationships. One recent study found that around half of YAs experience sexual dysfunction after diagnosis, which persists for at least two years [30]. However, research from the clinician perspective suggests providers inconsistently identify sexuality as an unmet need [31]. Our study demonstrates the relatively high unmet need for support with sexuality and sexual functioning among YAs compared to other domains and should motivate providers to address this gap. Expert consensus suggests that early initiation of discussion regarding sexual health counselling is important and that peer support may be an effective intervention for this population [32].

Similar to the previous findings in adults with cancer, the latent class analysis identified three classes of participants distinguished by level of supportive care need [25]. However, in this study, where we further explored the responses in each class, we found the degree of unmet need in each class ranged from none to moderate rather than low to high. While in general the unmet needs were not high, participants tended to have a similar degree of unmet need across domains. This suggests that resources should be targeted to those with supportive care needs in a holistic, multidisciplinary approach. One study found that using a conversation aid called a ‘Snapshot’ with adolescents and YAs helped identify psychosocial issues [33]. This could be a useful tool to identify supportive care needs across domains in this population.

The relationship between diagnosis and level of supportive care need could not be explored in-depth due to the small numbers in each group. However, the proportion of patients in each class for most diagnoses followed a similar pattern with the highest proportion of patients in the no-need class. This concurs with the findings of a previous systematic review that found that unmet supportive care needs did not differ by cancer type when included in mixed studies [34]. However, treatment status made a big difference for the level of supportive care need where the majority of those on treatment had unmet needs compared to the minority of those on follow-up. This also corroborated the results of the previous review, which found that patients on treatment had the highest unmet supportive care needs [34]. However, in the multivariable model, cancer type and treatment status were no longer significantly associated with level of supportive care need. It is also interesting to note that 8% of patients reported they did not know the intent of their treatment. It is difficult to interpret the reason patients reported unknown treatment intent, but this may have contributed to an observed higher information and psychological need in this group.

In multivariable analysis, the moderate-need class was independently associated with lower HRQoL, more helplessness, and more unmet service needs. This suggests that service needs do indeed play a role in unmet supportive care needs. While causality cannot be determined due to the cross-sectional design of the study, it is reasonable to expect that improving access to services would reduce the degree of unmet supportive care need for those with moderate need. However, this finding also suggests that HRQoL and helplessness play a role in unmet supportive care needs regardless of access to services. This implies that increasing services alone will not resolve all supportive care needs. We hypothesise that this may be the case because the SCNS measures issues that services may not consistently resolve. For example, ‘changes to daily routine and lifestyle’ may occur regardless of professional support due to cognitive or functional changes. Another example is ‘fatigue’, where there is uncertainty around effective interventions for YAs [35]. Addressing these issues will rely on reducing the initial impact of cancer and its treatment by finding kinder treatments and improving early diagnosis. Including patient-reported outcomes important to YAs in clinical trials and focusing on this specific population in analysis will help identify treatments with lesser impact on the issues important to this population.

### Limitations

The low response to this study should be taken into consideration when evaluating the results of this exploratory analysis. Low response is more common in studies focusing on adolescents and YAs, and it is recognised that recruitment in this population takes considerable resources [36]. Future researchers could employ a combined approach of in clinic and postal invitations to increase the proportion of responses. The low response may have introduced a response bias, which includes the overrepresentation of white females with breast cancer. However, breast cancer is the most common cancer among YAs, particularly between 35 and 39 years, which may account in part for the high proportion in this study [37]. Incomplete data further reduced the sample size in the multiple regression model. The inclusion of many different cancer types allowed us to explore the supportive care needs of YAs across diagnoses. However, broad diagnostic categories and relatively low numbers in some groups limited our ability to explore differences by cancer type. The diagnoses and treatments presented here may also suffer from some level of inaccuracy as participants self-reported the information.

## 5. Conclusions

YAs with cancer need additional psychological support, particularly for fear of cancer recurrence and uncertainty. Sexual needs have high importance relative to other domains in YAs and deserve special attention as this is often overlooked in routine care. Patients with unmet supportive care needs should be offered holistic care across the supportive care domains. Improving access to support services will likely reduce supportive care needs, particularly by targeting YAs with moderate need. However, some needs identified in the SCNS may not be effectively resolved by current services or interventions. Future studies should further explore the relationship between supportive care needs, HRQoL, and illness cognitions in specific supportive care domains and longitudinally to better understand causation.

## Figures and Tables

**Table 1:** Summary of patient-reported demographic and clinical characteristics in total sample and stratified by level of supportive care need.

	Total YAs (N = 317)	No Need (N = 168)	Low Need (N = 98)	Moderate Need (N = 51)
	Mean (SD)	Mean (SD)	Mean (SD)	Mean (SD)
**Mean age at diagnosis in years**	33.3 (4.2)	33.0 (4.3)	33.6 (4.1)	33.7 (4.1)
**Mean current age in years**	36.2 (4.5)	36.2 (4.6)	36.2 (4.6)	36.0 (4.3)
**Years from diagnosis**	2.9 (1.6)	3.2 (1.6)	2.6 (1.7)	2.3 (1.5)
	Frequency (column %)	Frequency (row %)	Frequency (row %)	Frequency (row %)
**Gender**				
Female	219 (69.1)	106 (48.4)	74 (33.8)	39 (17.8)
Male	98 (30.9)	62 (63.3)	24 (24.5)	12 (12.2)
**Ethnicity**				
White	272 (85.8)	147 (54.0)	88 (32.4	37 (13.6)
Asian/Asian British	26 (8.2)	13 (50.0)	6 (23.1)	7 (26.9)
Mixed/Multiple ethnic groups	12 (3.8)	6 (50.0)	2 (16.7)	4 (33.3)
Black/African/Caribbean/Black British	3 (1.0)	1 (33.3)	1 (33.3)	1 (33.3)
Other ethnic group	4 (1.3)	1 (25.0)	1 (25.0)	2 (25.0)
**Educational attainment**				
University	205 (64.7)	109 (53.2)	60 (29.3)	36 (17.6)
College/diploma	59 (18.6)	27 (45.8)	22 (37.3)	10 (16.9)
Secondary school	31 (9.8)	20 (64.5)	7 (22.6)	4 (12.9)
Vocational qualification	16 (5.05)	9 (56.2)	6 (37.5)	1 (6.25)
Primary school	2 (0.6)	1 (50.0)	1 (50.0)	0 (0.0)
Other	4 (1.3)	2 (50.0)	2 (50.0)	0 (0.0)
**Diagnosis**				
Breast cancer	102 (32.2)	39 (38.2)	41 (40.2)	22 (21.6)
Testicular cancers	47 (14.8)	31 (66.0)	13 (27.7)	3 (6.4)
Gynaecological cancers	45 (14.2)	21 (46.7)	15 (33.3)	9 (20.0)
Haematological cancers	37 (11.7)	20 (54.1)	11 (29.7)	6 (16.2)
Sarcomas	26 (8.2)	23 (88.5)	2 (7.7)	1 (3.8)
Head & neck cancers *	23 (7.3)	16 (69.6)	5 (21.7)	2 (8.7)
Gastrointestinal cancers	14 (4.4)	5 (35.7)	6 (42.9)	6 (21.4)
Melanoma	11 (3.5)	9 (81.8)	1 (9.1)	1 (9.1)
Other	12 (3.8)	4 (33.3)	4 (33.3)	4 (33.3)
**Treatments received (non-exclusive)**				
Surgery	250 (78.9)	135 (54.0)	78 (31.2)	37 (14.8)
Chemotherapy	184 (58.0)	81 (44.0)	68 (33.7)	35 (19.0)
Radiotherapy	144 (45.4)	71 (49.3)	48 (33.3)	25 (17.4)
Hormone therapy	66 (20.8)	29 (43.9)	26 (39.4)	11 (16.7)
Clinical trial therapy	34 (10.7)	15 (44.1)	12 (35.3)	7 (20.6)
Complementary therapy	29 (9.2)	11 (37.9)	11 (37.9)	7 (24.1)
Targeted therapy	28 (8.8)	12 (42.9)	11 (39.3)	5 (17.9)
Immunotherapy	19 (6.0)	7 (36.8)	8 (42.1)	4 (21.1)
Active surveillance	13 (4.1)	9 (69.2)	2 (15.4)	2 (15.4)
Stem cell transplant	7 (2.2)	6 (85.7)	1 (14.3)	0 (0.0)
Other	29 (9.2)	15 (51.7)	10 (34.5)	4 (13.8)
**Current treatment status**				
On follow-up	242 (76.3)	150 (62.0)	65 (26.9)	27 (11.2)
On treatment	75 (23.7)	18 (24.0)	33 (44.0)	24 (32.0)
**Current treatment intent**				
Curative	244 (77.0)	137 (56.1)	75 (30.7)	32 (13.1)
Palliative	46 (14.5)	18 (39.1)	15 (32.6)	13 (28.3)
Unknown	25 (7.9)	12 (48.0)	7 (28.0)	6 (24.0
Missing	2 (0.6)	1 (50.0)	1 (50.0)	0 (0.0)

YA, young adult; SD, standard deviation; N, number of observations * head and neck cancer comprised of thyroid cancer and other malignancies in the head and neck not further defined.

**Table 2:** Overall domain scores and proportion with unmet need (domain score >2) in each supportive care need domain and single item stratified by predicted latent class.

Domain	Total YAs (N = 317)		No Need (N = 168)		Low Need (N = 98)		Moderate Need (N = 51)	
	Median DS (IQR)	Number with DS >2 (%)	Median DS (IQR)	Number with DS >2 (%)	Median DS (IQR)	Number with DS >2 (%)	Median DS (IQR)	Number with DS >2 (%)
**Psychological** **(22 items)**	1.8 (1.2–2.7)	133 (42.0%)	1.2 (1.0–1.6)	0 (0.0)	2.5 (2.2–3.0)	83 (84.7)	3.3 (2.9–3.6)	50 (98.0)
**Health system and information** **(15 items)**	1.5 (1.0–2.3)	107 (33.8%)	1.1 (1.0–1.4)	6 (3.6)	2.1 (1.7–2.5)	50 (51.0)	3.2 (2.7–3.7)	51 (100.0)
**Physical and daily living** **(7 items)**	1.3 (1.0–2.1)	87 (27.4%)	1.0 (1.0–1.3)	0 (0.0)	2.1 (1.6–2.6)	56 (57.1)	2.6 (1.8–3.3)	31 (60.8)
**Patient care and support** **(8 items)**	1.3 (1.0–2.0)	59 (18.6%)	1.0 (1.0–1.3)	1 (0.6)	1.6 (1.1–2.0)	7 (7.1)	2.6 (2.3–3.3)	51 (100.0)
**Sexuality** **(3 items)**	1.7 (1.0–3.0)	115 (36.3%)	1.0 (1.0–1.7)	19 (11.3)	2.3 (1.1–3.3)	53 (54.1)	3.7 (2.7–4.2)	43 (84.3)
**Talking to other people** **(1 item)**	2.0 (1.0–3.0)	103 (32.6)	1.0 (1.0–2.0)	17 (10.1)	3.0 (2.0–3.0)	52 (53.1)	3.0 (2.0–4.0)	34 (68.0)
**Changes in others’ attitudes or behaviour towards you** **(1 item)**	1.0 (1.0–3.0)	94 (29.7)	1.0 (1.0–1.0)	11 (6.5)	2.0 (1.0–3.0)	47 (48.)	3.0 (2.0–4.0)	36 (70.6)
**Financial concerns** **(1 item)**	1.0 (1.0–3.0)	93 (29.3)	1.0 (1.0–1.0)	11 (6.5)	2.0 (1.0–3.0)	48 (49.0)	3.0 (2.0–5.0)	34 (66.7)
**Transport** **(1 item)**	1.0 (1.0–2.0)	42 (13.2)	1.0 (1.0–1.0)	2 (1.2)	2.0 (1.0–2.0)	13 (13.3)	3.0 (2.0–3.0)	27 (52.9)

IQR, interquartile range; DS, domain score; N, number of observations.

**Table 3:** Ten most common unmet supportive care needs stratified by level of supportive care need.

	10 Most Common Unmet Needs in No-Need Class			10 Most Common Unmet Needs in Low-Need Class			10 Most Common Unmet Needs in Moderate-Need Class		
	Number (%)	Item	Domain	Number (%)	Item	Domain	Number (%)	Item	Domain
1	60 (35.7)	Fears about the cancer returning	Psychological	80 (81.6)	Fears about the cancer returning	Psychological	49 (96.1)	Uncertainty about the future	Psychological
2	35 (20.8)	Uncertainty about the future	Psychological	77 (78.6)	Uncertainty about the future	Psychological	43 (84.3)	Fears about the cancer returning	Psychological
3	27 (16.1)	Anxiety	Psychological	74 (75.5)	Feelings of sadness	Psychological	42 (82.4)	Feelings of sadness	Psychological
4	24 (14.3)	Fears about the cancer spreading	Psychological	73 (74.5)	Anxiety	Psychological	42 (82.4)	Fears about the cancer spreading	Psychological
5	23 (13.7)	Feelings of sadness	Psychological	70 (71.4)	Feeling down or depressed	Psychological	42 (82.4)	Learning to feel in control of your situation	Psychological
6	23 (13.7)	To talk to someone who has been through a similar experience	Health system and information	68 (69.4)	Fears about the cancer spreading	Psychological	42 (82.4)	Keeping a positive outlook	Psychological
7	22 (13.1)	Lack of energy and tiredness	Physical and daily living	62 (63.3)	Lack of energy and tiredness	Physical and daily living	42 (82.4)	Access to professional counselling if you, family or friends need it	Health system and information
8	21 (12.5)	Feeling down or depressed	Psychological	59 (60.2)	Learning to feel in control of your situation	Psychological	41 (80.4)	Finding meaning in this experience	Psychological
9	19 (11.3)	Changes in sexual feelings	Sexuality	56 (57.1)	Keeping a positive outlook	Psychological	41 (80.4)	Changes to your usual routine and lifestyle	Psychological
10	19 (11.3)	Changes in your sexual relationships	Sexuality	55 (56.1)	Feelings about death and dying	Psychological	41 (80.4)	Changes in your sexual relationships	Sexuality
							41 (80.4)	Making the most of your time	Psychological

YA, young adult.

**Table 4:** Summary of psychosocial and service use outcomes in total sample and stratified by level of supportive care need.

	Total YAs (N = 317)	No Need (N = 168)	Low Need (N = 98)	Moderate Need (N = 51)
	Median (IQR)	Median (IQR)	Median (IQR)	Median (IQR)
* EORTC-QLQ-C30 summary score (N = 310)	89.0 (4.8–95.5)	94.4 (89.2–98.1)	78.9 (65.4–89.3)	71.7 (58.4–82.9)
**^+^ Acceptance (N = 314)**	16.0 (13.0–19.0)	17.0 (15.0–20.0)	14.0 (12.0–16.0)	13.0 (12.0–16.0)
**^+^ Helplessness (N = 314)**	8.0 (6.0–11.0)	6.0 (6.0–8.0)	10.0 (7.0–13.0)	12.0 (9.0–14.5)
**^+^ Perceived benefits (N = 314)**	18.0 (13.0–22.0)	18.0 (13.0–22.0)	18.0 (13.0–22.0)	17.0 (12.0–22.0)
**^#^ Unmet service needs (N = 287)**	2.0 (0.0–4.0)	1.0 (0.0–2.0)	3.0 (1.0–5.0)	5.0 (2.0–6.0)
	Frequency (column %)	Frequency (row %)	Frequency (row %)	Frequency (row %)
**^ⱡ^ Anxiety**				
**Score ≤ 8**	167 (52.7)	117 (70.1)	39 (23.4)	11 (6.59)
**Score > 8**	150 (47.3)	51 (34.0)	59 (39.3)	40 (26.7)
**^ⱡ^ Depression**				
**Score ≤ 8**	252 (79.5)	159 (63.1)	66 (26.2)	27 (10.7)
**Score > 8**	65 (20.5)	9 (13.8)	32 (49.2)	24 (36.9)

YA, young adult; IQR, interquartile range; N, number of observations; * European Organisation for Research and Treatment of Cancer Quality of Life Core Module score summarising all scales except for the financial impact scale and global quality of life; ^ⱡ^ Scale from the Hospital Anxiety and Depression Scale; ^+^ Scale from the Illness Cognitions Questionnaire; ^#^ Total number of unmet service needs from service need questionnaire.

**Table 5:** Univariable models with covariates significantly associated with level of supportive care need.

		Class Comparison	OR	DF	OR CI	*p*-Value
**In follow-up/on treatment (ref)**		low/no need	0.24	12	(0.09, 0.59)	0.005 **
		moderate/no need	0.10	12	(0.04, 0.25)	<0.001 **
**Non-breast diagnosis/breast diagnosis (ref)**		low/no need	2.91	12	(1.42, 5.99)	0.007 **
		moderate/no need	2.34	12	(1.07, 5.12)	0.035 *
**Non-white ethnicity/white ethnicity (ref)**		low/no need	0.65	12	(0.20, 2.11)	0.444
		moderate/no need	3.11	12	(1.26, 7.68)	0.018 *
**Treatment intent**	Palliative/curative (ref)	low/no need	1.32	10	(0.44, 3.91)	0.587
		moderate/no need	3.23	10	(1.21, 8.63)	0.024 *
	I don’t know/curative (ref)	low/no need	1.00	10	(0.26, 3.89)	0.996
		moderate/no need	2.24	10	(0.65, 7.68)	0.175
**Years from diagnosis**		low/no need	0.82	12	(0.67, 1.00)	0.050
		moderate/no need	0.68	12	(0.53, 0.89)	0.008 **
**Chemotherapy received/no chemotherapy (ref)**		low/no need	3.67	12	(1.64, 8.23)	0.004 **
		moderate/no need	2.03	12	(0.96, 4.28)	0.060
**EORTC-QLQ-C30 summary score ^‡^**		low/no need	0.87	12	(0.84, 0.91)	<0.001 **
		moderate/no need	0.85	12	(0.81, 0.89)	<0.001 **
**Anxiety ^ⱡ^ >8/** **Anxiety ≤8 (ref)**		low/no need	3.26	12	(1.64, 6.51)	0.003 **
		moderate/no need	16.54	12	(4.50, 60.83)	0.001 **
**Depression ^ⱡ^ >8/** **Depression ≤8 (ref)**		low/no need	8.85	12	(3.22, 24.33)	0.001 **
		moderate/no need	20.38	12	(6.48, 64.08)	<0.001 **
**Helplessness ^+^**		low/no need	1.72	12	(1.44, 2.06)	<0.001 **
		moderate/no need	1.98	12	(1.61, 2.45)	<0.001 **
**Acceptance ^+^**		low/no need	0.82	12	(0.74, 0.90)	0.001 **
		moderate/no need	0.80	12	(0.72, 0.88)	<0.001 **
**Unmet service needs ^#^**		low/no need	1.31	12	(1.13, 1.45)	0.002 **
		moderate/no need	1.70	12	(1.51, 2.00)	<0.001 **

OR, odds ratio; DF, degrees of freedom; CI, confidence interval; ^‡^ European Organisation for Research and Treatment of Cancer Quality of Life Core Module score summarising all scales except the financial impact scale and global quality of life; ^+^ Scale from the Illness Cognitions Questionnaire; ^ⱡ^ Scale from the Hospital Anxiety and Depression Scale; ^#^ Total number of unmet service needs from service need questionnaire; * *p*-value significant to 0.05 level; ** *p*-value significant to 0.01 level.

**Table 6:** Final multivariable regression showing covariate relationships with level of supportive care need.

Variable	Class Comparison	OR	OR CI	*p*-Value
**EORTC-QLQ-C30 Summary score ^‡^**	low/no need	0.92	(0.88, 0.98)	0.012 *
	moderate/no need	0.90	(0.85, 0.96)	0.008 **
**Helplessness ^+^**	low/no need	1.34	(1.04, 1.73)	0.030 *
	moderate/no need	1.42	(1.03, 1.95)	0.035 *
**Unmet service needs ^#^**	low/no need	1.18	(0.97, 1.44)	0.082
	moderate/no need	1.57	(1.21, 2.04)	0.005 **
**Acceptance ^+^**	low/no need	0.93	(0.81, 1.06)	0.202
	moderate no need	0.86	(0.73, 1.02)	0.074
**Model Characteristics: AIC 1141.547 | BIC 1232.683 | Residual DF 6 | Observations 283**				

OR, odds ratio; DF, degrees of freedom; CI, confidence interval; AIC, Akaike’s Information Criteria; BIC, Bayesian Information Criteria (BIC); ^‡^ European Organisation for Research and Treatment of Cancer Quality of Life Core Module score summarising all scales except the financial impact scale and global quality of life; ^+^ Scale from the Illness Cognitions Questionnaire; ^#^ Total number of unmet service needs from service need questionnaire; * *p*-value significant to 0.05 level; ** *p*-value significant to 0.01 level.

## Data Availability

Data from this study are not available, as participants did not consent for reuse in future studies.

## Supplementary Materials

The following are available online at https://www.mdpi.com/article/10.3390/jcm10194449/s1, Table S1: Service need questionnaire, Material S2: Detailed information regarding model fit and selection in latent class analysis.
