# Supplementary material for: Describing Unmet Supportive Care Needs among Young Adults with Cancer (25–39 Years) and the Relationship with Health-Related Quality of Life, Psychological Distress, and Illness Cognitions
Source: J Clin Med. 2021 Sep 28;10(19):4449. doi: 10.3390/jcm10194449 (PMC8509768; doi:10.3390/jcm10194449)
Supplement: Supplementary file 1 [file jcm-10-04449-s001.zip › Supplementary Material S1. Service Needs Questionnaire.pdf]

Supplementary Material Table S1. Service needs questionnaire.

	Please indicate whether you need(ed) any of the following services during or after your treatment for cancer and whether you used them.	**Did you need this service?**		**Did you use the service?**	
		**Yes**	**No**	**Yes**	**No**
a.	Physiotherapist	❑	❑	❑	❑
b.	Pain management expert	❑	❑	❑	❑
c.	Psychologist, mental health worker (e.g. managing anxiety or worry)	❑	❑	❑	❑
d.	Spiritual or religious counselor	❑	❑	❑	❑
e.	Social worker/occupational therapist	❑	❑	❑	❑
f.	Financial expert (e.g. advice to help figure out payments)	❑	❑	❑	❑
g.	Psychosexual therapist (e.g. advice about sexual issues)	❑	❑	❑	❑
h.	Peer support group (online or in person)	❑	❑	❑	❑
i.	Second opinion about treatment	❑	❑	❑	❑
j.	Dietician	❑	❑	❑	❑
k.	Fertility counseling	❑	❑	❑	❑
m.	Help with childcare	❑	❑	❑	❑
n.	Help with care for parents	❑	❑	❑	❑
o.	Psychological support for partner/spouse	❑	❑	❑	❑
p.	Palliative care counseling or support	❑	❑	❑	❑
q.	Complementary and alternative medicine	❑	❑	❑	❑
