# Supplementary material for: Describing Unmet Supportive Care Needs among Young Adults with Cancer (25–39 Years) and the Relationship with Health-Related Quality of Life, Psychological Distress, and Illness Cognitions
Source: J Clin Med. 2021 Sep 28;10(19):4449. doi: 10.3390/jcm10194449 (PMC8509768; doi:10.3390/jcm10194449)
Supplement: Supplementary file 1 [file jcm-10-04449-s001.zip › Supplementary Material S2. Details of model fit and selection.pdf]

Supplementary Material S2. Detailed information regarding model fit and selection in latent class analysis.

Latent class models with increasing numbers of classes were fit from a 1-class model to a 5-class model. Model fit was assessed using the exact test due to small expected numbers. Model selection was based on minimising the Bayesian Information Criteria (BIC) and Akaike's Information Criteria (AIC), increasing the entropy and ease of interpretation (i.e. the classes make sense from a rational perspective).

To test the assumption of conditional independence we examined the associations between domains within classes using Fisher's exact test. We adjusted for multiple comparisons (30 domain pairs compared) using the Bonferroni correction and considered p-values <0.002 to be statistically significant.

Both the AIC and BIC decreased from the 2-class model to the 3-class model indicating better fit (Table X). The AIC of the 4-class model decreased compared to the 3-class model, however, the BIC increased and entropy decreased. Previous literature suggests the BIC is a more robust indicator of class structure (1). The 4-class model also differentiated individuals in the low need class into two groups where some had need in the sexual domain. This domain, however, is limited to three items and was therefore felt to be unreliable in differentiating individuals alone and the existence of need only in the sexual domain seemed unlikely. Therefore, as the 3-class model had a lower BIC, higher entropy and was more coherent, the 3-class model was selected.

Table x shows the probability of dichotomised domain score given class membership. Values close to zero or one indicate good differentiation, while values close to 0.5 suggest class membership does not determine the response for that domain and the response can be random. The 3-class model showed good differentiation in most domains particularly in the high and low need classes.

References

1. Nylund, Karen L.;Asparouhov, T.; Muthen BO. Deciding On the Number of Classes In Latent Class Analysis and Growth Mixture Deciding on the Number of Classes in Latent Class Analysis and Growth Mixture Modeling : A Monte Carlo Simulation Study. 2015;(February):37–41.

Table 1. Latent class model selection criteria.

Classes	Exact Test	AIC	BIC	Entropy
1	0.0	1939.083	1957.878	3.043
2	0.00703	1508.148	1549.496	2.356
3	0.97187	1481.616	1545.517	2.290
4	1.0	1478.886	1565.341	2.259
5	1.0	1486.841	1595.849	2.254

AIC**,** Akaike's Information Criteria; BIC**,** Bayesian Information Criteria

Table 2. Domain response probabilities given class membership.

Domain	Class	Probability of domain score ≤2	Probability of domain score >2
**Psychological**	1	1.0000	0.0000
	2	0.1534	0.8466
	3	0.0202	0.9798
**Health System & Information**	1	0.9502	0.0498
	2	0.5505	0.4495
	3	0.0000	1.0000
**Physical and daily living**	1	0.9931	0.0069
	2	0.4390	0.5610
	3	0.3901	0.6099
**Patient care and support**	1	0.9923	0.0077
	2	0.8791	0.1209
	3	0.1960	0.8040
**Sexuality**	1	0.8719	0.1281
	2	0.5232	0.4768
	3	0.1318	0.8682
